# Psoriatic Arthritis Priority Setting Partnership: patient- and clinician-informed considerations for future UK health service delivery

**DOI:** 10.1093/rheumatology/keae680

**Published:** 2024-12-20

**Authors:** Lija James, Louise Hailey, Christine Bundy, Helen Burstow, David Chandler, Russ Cowper, Philip Helliwell, Lucille Joannes, Andy Kelly, Bronagh Kennedy, Suzannah Kinsella, Helen McAteer, Sandeep Mukherjee, Jon Packham, Elspeth Wise, Helen Young, Emma Dures, Laura C Coates

**Affiliations:** Nuffield Department of Orthopaedics, Rheumatology and Musculoskeletal Sciences (NDORMS), University of Oxford, Oxford, UK; Nuffield Department of Orthopaedics, Rheumatology and Musculoskeletal Sciences (NDORMS), University of Oxford, Oxford, UK; School of Healthcare Sciences, Cardiff University, Cardiff, UK; Rheumatology Service, University Hospitals Dorset NHS Foundation Trust, Christchurch, UK; Psoriasis and Psoriatic Arthritis Alliance (PAPAA), St Albans, UK; Patient Research Partner, Innovation Health Manchester, Manchester, UK; Leeds Institute of Rheumatic and Musculoskeletal Medicine, University of Leeds, Leeds, UK; Patient Research Partner, Tunbridge Wells, UK; Patient Research Partner, Cardiff, UK; Patient Research Partner, London, UK; James Lind Alliance Adviser and Chair of the Steering Group, Tunbridge Wells, UK; Psoriasis Association, Northampton, UK; Rheumatology Service, University Hospitals Dorset NHS Foundation Trust, Christchurch, UK; Haywood Rheumatology Centre, The Haywood Hospital, Stoke-on-Trent, UK; Clinical Associate Professor in Rheumatology, Academic Unit of Population and Lifespan Sciences, University of Nottingham, Nottingham, UK; Talbot Medical Centre, General Practitioner, South Shields, UK; Centre for Dermatology Research, Manchester Academic Health Science Centre, The University of Manchester, Manchester, UK; School of Health and Social Wellbeing, University of the West of England, Bristol, UK; Nuffield Department of Orthopaedics, Rheumatology and Musculoskeletal Sciences (NDORMS), University of Oxford, Oxford, UK

**Keywords:** health service delivery, psoriatic arthritis, priority setting partnership, rheumatology

## Abstract

**Objectives:**

Little is known about the ideal service delivery model and shortcomings in patient experiences in the NHS for patients with psoriatic arthritis (PsA). The objective of this work was to identify unmet needs perceived within the current health service delivery model for PsA from the UK Psoriatic Arthritis Priority Setting Partnership (PsA-PSP).

**Methods:**

An online survey was conducted in 2020 and distributed to people with PsA, their carers and clinicians to identify research priorities in PsA. The participants were asked to submit three questions unanswered in PsA research. A proportion of submissions related to health service delivery were identified, which were deemed as out of scope for the main PsA-PSP but nevertheless important to report. Content analysis was used to analyse these submissions separately.

**Results:**

We reviewed 138 submissions that were not related to the James Lind PSP and research priorities in PsA. Among these, 118 (85.5%) were focused on health service delivery and were classified into five main themes: rheumatology service, primary care navigation, education, holistic care, and ethnicity, diversity and inclusion. Further analysis within the rheumatology service theme revealed additional sub-themes that emphasized integrating multidisciplinary services, improving access to advice lines and ensuring fair access to treatments.

**Conclusion:**

The five key themes provide valuable insights into the important areas of interest within health service delivery in the UK. By understanding these themes, policymakers, healthcare providers and researchers can better prioritize their efforts and address the specific care needs of people with PsA, their care providers and clinicians.

Rheumatology key messages

- Multi-disciplinary clinics are a priority that may improve timely diagnosis and provide more personalized care.
- Pain management, physical therapy and non-pharmacological interventions are recommended for high-quality care and patient satisfaction.
- Patient education and access to trained allied health professionals will optimize the delivery of care services.

## Introduction

Healthcare provision falls short of meeting the needs of some groups of patients, as indicated by the UK national early inflammatory arthritis audit of 2023 (NEIAA 2023) [1]. People with long-term conditions across the UK are not always best served by how healthcare services are designed, delivered and managed. A 2015 healthcare systems framework identified six key domains in healthcare delivery. Domain 4 of the framework highlights the importance of incorporating patient preferences in healthcare delivery [2].

The James Lind Alliance (JLA) was established in 2004 to support healthcare research priorities through the implementation of Priority Setting Partnerships (PSP). A PSP involves collaboration among all stakeholders, including individuals living with health conditions, their caregivers, relatives, friends and clinicians [3]. In 2021, a PSP workshop was conducted to identify and prioritize the top 10 research questions related to psoriatic arthritis (PsA). Although the results of this survey were published, the steering group noted that many significant questions submitted were focused on health service delivery. This report presents findings from the UK PsA-PSP surveys, highlighting unmet needs in health service delivery and outlining key recommendations for a future health service delivery model for psoriatic arthritis.

## Methods

### Study design and data collection

The PsA-PSP involved three stages as established by the JLA methodology, comprising surveys, focus groups and interviews. Firstly, a survey was carried out online with individuals affected by PsA, caregivers and clinicians to identify significant unanswered questions in PsA research. Originally intended to be conducted on paper and online, the format was adjusted due to the SARS-CoV2 pandemic in early 2020, leading to an online-only approach. The survey was conducted using the Jisc online survey platform from a leading UK digital, data and technology agency focused on tertiary education and research.

The survey was widely promoted to people living with PsA, their families and carers through our collaboration with organizations, such as the Psoriasis Association, and was conducted through email, newsletters, websites and social media platforms. The survey was circulated for 4 months between June and October 2020. As this was widely shared via different media, a response rate to the survey cannot be estimated.

Participants were asked to submit up to three questions they thought were the most important unanswered questions about PsA. In the second stage, the questions were reviewed with existing evidence to identify ‘true uncertainties’ and were then categorized into ‘indicative questions’ representing the main themes. Subsequently, a second online survey was conducted to prioritize the ‘true uncertainties’ based on their significance. Finally, a workshop was held, involving individuals living with PsA and clinician stakeholders, to determine the top 10 research priorities.

In the final workshop for the PsA-PSP, the steering group reached a consensus on the ‘top 10’ unmet needs in PsA research. The ‘Psoriatic Arthritis PSP Top 10’ was published in August 2021 [4]. Patient and public involvement and engagement members were part of the steering committee. The full JLA methodology is detailed in the protocol (https://www.jla.nihr.ac.uk/documents/psoriatic-arthritis-psp-protocol/24155).

The steering committee identified a group of responses that are not directly related to PSP and PsA research priorities, but rather to health service delivery. As a result, the committee recommended that these responses be analysed separately, as they are considered important to report on.

### Analysis

Content analysis is an established methodology within qualitative research [5–7]. Previous studies have used a hybrid content analysis to analyse open-ended responses related to rheumatology consultation [8], inflammatory arthritis [9] and telemedicine appointments in the COVID-19 pandemic [10]. The responses that did not relate to PSP/PsA research priorities were downloaded into an Excel sheet. Each submission was given a unique identifier as shown in Table 1 and was allocated to one of three groups: (i) health service delivery questions, (ii) out-of-scope questions, and (iii) responses that did not ask a question. The responses that were not associated with a specific question and out-of-scope for PsA were excluded from the analysis (Supplementary Table S1, available at *Rheumatology* online). Submissions under the health services group (group 1) were further analysed in this report.

**Table 1.** Open codes from the submissions relating to health service delivery

A lack of help to manage pain and fatigue	Feeling unheard	Remote consultations
Access	GP knowledge	Rheumatology knowledge
Acquiring strategies	Guidelines	Screening
Awareness	Health care provider’s training needs	Self-help
Benefits	Holistic care	Skin
Combined clinics	Latest evidence	Support
Consultation process	Misdiagnosis and missed opportunities	symptom onset to specialist care
Coping with symptoms	Monitoring	Team support
Cost (drugs)	Nails	Team working/MDT
Delays	NICE	Treatment
Diagnosis	Patient information	Understanding from clinicians
Diet	Poor communication	Understood by others
Diversity and inclusion	Primary *vs* secondary care	validation and reassurance
Exercise	Psychology	Ways of coping
Fast and easy access to expertise	Public health messages	Weighing up treatment options
Fear about the future	Referral	Whole team support

GP: general practitioner; MDT: multidisciplinary team; NICE: National Institute for Health and Care Excellence.

A hybrid qualitative content analysis was used to code data. Analysis began with the open coding of participants’ written words or sentences. Codes that shared a similar meaning into sub-categories were grouped. The number of sub-categories was then reduced by integrating those that were conceptually similar, and re-grouping them under higher-level main categories, creating a two-level hierarchy [8]. Two members of the research team independently conducted this phase of the analysis. A steering committee subgroup reviewed the allocation process and ensured their interpretations were consistent. The total number of responses will not sum to the total responses (i.e. 138) because some submissions were sorted into more than one category. Categories were assigned based on recurring themes in the responses.

## Results

Of the original 999 submissions, 69% of the respondents were people living with PsA, 15% were friends, relatives or carers of someone affected by PsA, and clinicians submitted the remaining 16%. The survey contained 138 submissions that were not related to the JLA PSP and research priorities in PsA. We found that 86% (118 out of 138) of them focused on aspects related to health service delivery, 9% (13 of 138) were not phrased as a question and 5% (7 of 138) were considered out-of-scope for PsA (See Supplementary Table S1, available at *Rheumatology* online).

Upon further analysis, five themes were identified within the health service delivery group. These include rheumatology service, primary care navigation, education, holistic care, and ethnicity, diversity and inclusion. The findings have been condensed and tabulated in Table 2.

**Table 2.** Summary of categories identified through content analysis

No.	Theme	No. of submissions, *n* responses or *n* (%)	Selected example of submissions
1	Rheumatology service	44 responses	
1a	Rheumatology service: Integration of multidisciplinary clinics	19 (43)	Combined care with Dermatologist or Gastroenterologist—how joined up is it for a patient? Why are PsA patients treated by two complete separate specialties who don’t work together—e.g. rheumatology and dermatology. Why not an MDT? Should Rheum/derm/gastro combine, i.e. immunological conditions to better manage patients’ symptoms and improve treatments.
1b	Rheumatology service: Advice line and urgent care	15 (34)	
1c	Rheumatology service: Access to treatment	10 (23)	BSR guidance is not in keeping with NICE—where one DMARD can be used if poor prognostic factors. Can both be aligned? Is the rationing of biologic drugs costing the NHS more in the long run? Access to funding. Why are PsA sufferers always made to wait longer for NICE to approve and then fund new treatment? This is usually a year or so after a drug has been approved for RA. What is frustrating is this includes drugs that have been developed specifically targeting PsA (‘…’ using secukinumab as an example). We often feel like the poor relation to RA patients. What are the qualifying criteria for patients with PsA to be eligible for newer generation treatments [biologics (e.g. biosimilars)] on the NHS and do these vary by postcode?
2	Primary care navigation	33 responses	Time delays to see specialist are a problem. From initial presentation of arthritic symptoms to GPs, how long does it take to diagnose those patients that have psoriatic arthritis? Overall, I feel the biggest problem seems to be the referral from GP to rheumatology and I think that’s where lots of work needs to be done.
3	Education	39 responses	
3a	Education: Primary care	29 (74)	Are GPs fully aware of symptoms of psoriatic arthritis and when they should refer a person for assessment? How to help doctors in general practice to differentiate inflammatory psoriatic arthritis from osteoarthritis? Many GPs are unaware of PsA. Always diagnose nail fungus! Better understanding and education required.
3b	Education: Secondary care	8 (21)	Should all HCPs train to manage skin symptoms? Why don’t dermatologists advise you of the risk of psoriatic arthritis? Training in the early detection of psoriatic arthritis in podiatrists. Why are so many of us faced with consultant rheumatologists who seem to be unaware of the very particular nature of PsA—e.g. that inflammation may not show up on a blood test?
3c	Education: Patient and public	2 (5)	Are you providing more widespread information such as leaflets and posters to help society ‘accept’ people who have this? Although it is a complex condition, why has it been so difficult for me to find out all about PsA and have had to do so much online research myself?
4	Holistic care	17 responses	
4a	Non-pharmacological	8 (47)	Why isn’t a more holistic approach to treatment taken, for example assessment and plans for physio dieticians? Mental health Rheumatology appointments only focus on medication and investigations. What emotional/mental health support is available through the NHS post diagnosis? Why rheumatologist or any doctor never ask patient about their diet?
4b	Wider impact of disease	9 (53)	Why don’t medical professionals talk about/ask me about other aspects of living with PsA? How do I get my doctor to take my pain seriously and help me come up with a plan for tackling it? My main frustration is that treatment and support is not at the same level as other chronic long-term conditions, for example diabetes.
5	Ethnicity, diversity and inclusion	2 responses	What culture/ethnicity specific communication service and support is there, e.g. Asians with limited English language, any regions with extra support from secondary care or is there access from PsA/psoriasis patient organizations? How does time to referral and time to treatment vary in remote and rural settings compared with urban—interested in inequalities in care.

The total number of responses will not sum to 118 because some submissions were valid in more than one category. Categories were assigned based on recurring themes in the responses. BSR: British Society for Rheumatology; GP: general practitioner; HCP: healthcare professional; MDT: multidisciplinary team; NHS: National Health Service; NICE: National Institute for Health and Care Excellence.

### Rheumatology service

There were 44 submissions related to rheumatology service, which were divided into the following subcategories.

#### (i) Integration of multidisciplinary clinics

A total of 43% (*n* = 19) of the responses were related to combined working across medical specialities. Respondents expressed a need for improved co-ordination and collaboration with other relevant medical specialities, including gastroenterologists, dermatologists and allied health professionals (AHPs). Within surveys, AHP represents the dedicated team of nurses, physiotherapists and pharmacists who collaborate closely with rheumatology physicians.

#### (ii) Advice line and urgent care

Approximately 34% (*n* = 15) of the responses highlighted the need for access to rheumatology helplines for inquiries, appointments or urgent matters. Urgent questions to the helplines, where they existed, were significantly delayed, and contacting a care provider for an urgent inquiry was challenging for some. In the opinion of most survey respondents, a robust and user-friendly helpline system was essential for efficient communication between patients and healthcare providers. AHPs are crucial in assessing and prioritizing patient inquiries, ensuring that patients with urgent needs receive prompt attention. The lack of AHP services may have caused delays.

#### (iii) Access to treatment

A range of submissions 23% (*n* = 10) recounted issues around medication access. Responses reflected delayed or limited access to certain biologic treatments for patients with PsA. If they had severe enough skin psoriasis, which falls under dermatologists’ jurisdiction, these biologics would have been offered to them. Additionally, the PSP highlighted the discrepancy in medication access, particularly biologics, across different regions within the UK. It was evident from the submissions that PsA treatments require a more equitable and consistent approach.

### Primary care navigation

There were 33 submissions regarding care navigation within primary care settings, particularly on delayed referral to secondary care. This delay was often attributed to a perceived lack of knowledge among primary care clinicians. Based on the responses, difficulties getting through to the ‘right specialist’ was a problem (*n* = 3).

### Education

A total of 39 respondents noted a need for increased education and awareness about PsA among clinicians, AHPs, patients and the public. Approximately 74% (*n* = 29) of respondents suggested that general practitioners should be educated on the importance of early diagnosis and referral to rheumatologists for timely treatment. However, 21% (*n* = 8) of respondents indicated that practitioners in secondary care settings require more education.

### Holistic care

This section reports on the submissions on two subcategories: non-pharmacological care (*n* = 8) and the wider impact of disease (*n* = 9). A range of non-pharmacological treatments were overlooked and not provided in clinics, namely interventions to address nutrition, exercise and weight management. Based on the feedback from respondents, it was reported that certain clinicians exhibited a reluctance to explore the adverse social, emotional and financial effects of the disease on affected individuals. Respondents described feeling unheard as some clinicians do not always consider the wider impact of disease ‘beyond the number of joints’. Respondents also reported a lack of healthcare services pertaining to pain management, mental health and physical rehabilitation therapies in rheumatology.

### Ethnicity, diversity and inclusion

There were two submissions regarding support for culture-specific communication needs and access to care in rural settings, highlighting the importance of addressing the specific needs of all ethnic groups and considering how to meet the needs of patients with protected characteristics at risk of poor health outcomes.

## Discussion

The present PSP analysis helps identify the limiting factors and pitfalls in health service delivery specific to PsA from the perspective of people living with the condition, their carers and clinicians. Several hospital trusts in the UK fail to meet the benchmark time between referral and appointment with a rheumatologist, according to the NEIAA 2023 [1]. Compared with previous years, there has been an increase in assessment delays, with only 39% of patients being seen within the recommended timeframe [1]. Additionally, people living with PsA typically experience symptoms for a longer period before being referred to secondary care compared with those with rheumatoid arthritis [11]. A timely diagnosis and treatment can prevent irreversible joint damage in people with PsA and improve quality of life [12]. Targeted campaigns and educational seminars for clinicians could facilitate early diagnosis, improve referral efficiency and support a smoother patient journey in different care settings.

As per the findings of the PSP study, there is a growing need for multidisciplinary care to reduce delays, enhance patient navigation, and provide more personalized and timely care, as recommended by regulatory agencies such as the Care Quality Commission (CQC) [13]. Pain management, physical therapy, and non-pharmacological interventions, such as weight management and specifically graded exercise programmes, were deemed essential components of a comprehensive care approach for patients with PsA. Therefore, rheumatology clinics should incorporate these interventions into their treatment plans. Educating patients about chronic conditions has shifted from a compliance-driven approach to emphasizing patient empowerment and self-management [14].

The current study was not designed to determine the requirements for healthcare service delivery in the UK, which may lead to bias in the findings. This report serves as a subanalysis of the initial PsA-PSP survey, which sought to investigate the unmet needs in PsA research from the perspectives of all stakeholders involved.

The involvement of patients and public representatives is a notable strength, as they shared their personal experiences and perspectives. However, this survey method needs more clarity in specific responses, as about 9% of responses were identified as ‘not a question’. Although the survey was widely distributed, it is important to note that the respondents may not be fully representative of the patient population. The survey received fewer responses from men and ethnic minorities and was conducted entirely online. This could lead to a selection bias, as people with difficult-to-treat diseases or delayed diagnoses are more likely to complete the survey, potentially skewing the results. Similarly, those with limited information technology literacy and access to an online survey platform could have been disadvantaged. Therefore, a further study should be conducted specifically to address questions related to health service delivery in PsA. Nevertheless, the findings remain relevant for patients, clinicians and policymakers.

### Conclusion

The findings of this report suggest that there is a perceived unmet need in healthcare service delivery. Providing quality health service delivery to patients with PsA requires a patient-centred approach that addresses the multidimensional aspects of the disease. Integrated care optimizes treatment, which involves a collaborative team of healthcare professionals. In addition, engaging and empowering patients and caregivers increases transparency and accountability on issues of inequality in healthcare delivery services as evidence evolves. The recommendations in this report provide stakeholders with a vision for improving healthcare delivery for people living with PsA.

### Future work

As reported by care providers and care receivers, areas for development specifically related to health service delivery exist across primary and secondary care settings. These need further exploration when evaluating service innovations for people with long-term conditions.

## Supplementary Material

keae680_Supplementary_Data

## Data Availability

The data underlying this article will be shared on reasonable request to the corresponding author.
