# Supplementary material for: Psoriatic Arthritis Priority Setting Partnership: patient- and clinician-informed considerations for future UK health service delivery
Source: Rheumatology (Oxford). 2024 Dec 20;64(7):4335–40. doi: 10.1093/rheumatology/keae680 (PMC12212901; doi:10.1093/rheumatology/keae680)
Supplement: keae680_Supplementary_Data [file keae680_supplementary_data.docx]

SUPPLEMENTARY TABLE S1

Example of responses categorised into either not a question or out-of-scope for PsA

| Response submitted to survey | Category |
| --- | --- |
| all of the above! | Not a question |
| I think there are many important diseases to research than psoriatic arthritis | Not a question |
| Research agenda for PsA | Not a question |
| strategy trials | Not a question |
| I participated in the steering committee of the Psoriasis Association led Top 10 unanswered psoriasis research questions. I'm happy to help as and when with this project | Not a question |
| I think there are many important diseases to research than psoriatic arthritis | Not a question |
| Did your mother take any prescribed medication during pregnancy e,g, for morning sickness? | Not a question |
| Exploring juvenile PsA: is it different from adult-onset PsA? | Out of scope |
| Does it matter if someone who has a family history of psoriasis, but no personal rash is treated for Rheumatoid Arthritis instead? | Out of scope |
| How effective is sub-grouping e.g. axial vs peripheral vs monoarthritis vs RA like? Is there too much of a blur on the boundaries to make this workable? | Out of scope |
